# Twenty-Five Years of Charity

**Published:** 1901-01-05

**Authors:** 


### The Institutional Workshop.

### TWENTY-FIVE YEARS OF CHARITY.

The magnitude of the voluntary funds raised by the Mansion House for the cause of charity is not their only claim to the admiration of the world. The millions collected under the régime of successive Lord Mayors within the past quarter of a century tell a glorious story of human love and human sympathy. That the collection and distribution of these funds have involved the exercise of high organising ability, as well as an expenditure of no small labour, is sufficiently clear. But more wonderful from the human standpoint is the grand catholicity of the charitable efforts which have had as their centre the Mansion House of the City of London.

Within the twenty-five years that closed with 1900 the total sum raised by the agency of Mansion House Funds is equal to over four and three-quarter millions sterling—the round figures being £4,815,000. And except for a small proportion this great amount has been expended for purely charitable purposes. At such a result our Continental neighbours may well express their astonished admiration. Our friends across the Channel, indeed, would take no small pride unto themselves if they could point to a French charitable agency which, in the course of a quarter of a century, had collected and distributed a sum equivalent to more than a hundred and twenty million francs.

The money so freely given has not been dispensed solely within the boundaries of the United Kingdom. Its distribution has not even been confined to subjects of Her Majesty. In foreign lands, sufferers by great convulsions of Nature or from the horrors of war, from plague, from famine, and from persecution have had cause to turn with gratitude towards the City of London and to offer thanks to the people of an alien race who did not forget them in the day of trouble. A striking point in the history of Mansion House charitable efforts during the quarter of a century under review is the fact that the sums collected during the current year far exceed the total for any other year of the twenty-five. It is true that in 1900 the struggle in South Africa created special demands of a national character. That these demands have been nobly responded to is beyond dispute, and it is worthy of note that whereas in 1876 the sums raised through the agency of the Mansion House amounted to £73,000, the total for 1900 is £1,650,000.

While the needs of the distressed in far-off countries have been alleviated it cannot be alleged that those responsible for successive Mansion House Funds have forgotten the adage that "Charity begins at home." The record in connection with institutions for the treatment of disease in the Metropolis is instructive. It is frequently contended that the people of London might fairly be expected to contribute more liberally toward the support of their hospitals. By the agency of the Hospital Sunday Fund, which looms large in the history of Mansion House charitable effort, an admirable opportunity has been afforded to the churches of the Metropolis, and the extent to which they have availed themselves of this opportunity may be gathered from the fact that during the past twenty-five years they have contributed over a million pounds, the precise total being £1,017,769. In 1876 £27,692 was collected through this agency; while in 1900 the contributions have amounted to £52,716, or nearly double the former figures. Here is a record which the organisers may justly regard with pride. The collections of 1900 have twice been exceeded, in 1895 when the total was £62,758, and in 1899 when £54,942 was raised. But in view of the extraordinary demands on the purses of the charitable during the past year, the result in 1900 cannot be regarded as otherwise than highly satisfactory.

Whenever a great national disaster is marked in our recent history, the charitable efforts of the Mansion House are noted side by side. In one of the most famous books of reference the record of calamity is invariably accompanied by the significant note, "See also Mansion House Fund." When the appalling catastrophe to the *Princess Alice* in the Thames threw thousands of families into mourning and robbed hundreds of their bread-winners, over £38,000 was raised for the sufferers. The relief of the families of men lost in disasters to vessels of Her Majesty's Navy has invariably been the care of the occupants of the Chief Magistrate's chair. It is striking to observe that in the latest case of naval catastrophe the funds collected exceed in a most marked degree the total in any which went before. When, in 1878, three hundred men of H.M.S. *Eurydice* were drowned on a Sunday afternoon in March, in sight of the white cliffs of England, the Mansion House raised £5,713. For the families of the crew of two hundred and eighty of the *Atalanta*, "the ship that never returned," £9,897 was collected, while the loss of the *Serpent* with one hundred and seventy-two men in 1891, resulted in £3,231 being gathered. But when the awful catastrophe befell Sir George Tryon's flagship *Victoria* in 1893, and three hundred and fifty-nine seamen perished before the eyes of their comrades of the Mediterranean fleet, no less than £68,880 was contributed, a sum equivalent to about £200 for every gallant blue-jacket who went down.

Other notable funds organised for the relief of disasters and sufferings at home are £35,431 for Irish distress, £33,000 for the sufferers by the Abercarne Colliery explosion, £10,335 for the distress caused by the earthquake in Essex in 1884, £78,629 for the unemployed in London in 1886, £27,938 for the distress due to the devastation caused by the terrible storm in Essex in 1897, and £31,429 for the Jubilee dinners organised by H.R.H. The Princess of Wales. Here too must be mentioned the great fund of £1,028,650 raised in response to the patriotic appeal on behalf of the sufferers from the Transvaal war.

The famines which periodically devaste vast portions of our Indian Empire call forth not only the unwearied exertions of British administrators, but the deepest sympathy of the people of this country. Of the total amount collected through Mansion House Funds during the period under review, £1,455,000, or nearly one-third of the total, has been distributed for the benefit of the starving natives of India. In 1877, £515,200 was raised; in 1897, £550,423; and in 1900, despite the unprecedented demands on the nation's charity, the sum of £389,000.

Disasters and distress in our Colonies have resulted in the raising of £355,000 through Mansion House Funds during the quarter of a century. Of this sum considerably more than one-half stands to the credit of the present year, £54,000 having been collected for the sufferers by the great fire at Ottawa, and £178,000 for the Transvaal refugees. The latter is certainly exceptional, but is not the record a proof that the bonds of sympathy between the colonies and the Mother-country are being drawn closer as the years go by? In the various funds for colonial troubles are found £24,635 for the sufferers by the fire at St. John's, Newfoundland, in 1892, £12,118 for the victims of the hurricane in the Mauritius in the same year, and £53,450 for the assistance of those who suffered by the terrible storms in the West Indies a couple of years ago.

It has been stated above that the people of lands outside the Empire have been succoured in their distress by the charitable efforts of the Mansion House. The great fund raised for the relief of the French sufferers by the war of 1870 does not come within the scope of this article; but since 1875 £255,000 has been gathered to alleviate the woes of the victims in foreign countries of famine, war, persecution, and pestilence. For the persecuted Jews in Russia £108,809 was contributed in 1882, and in the same year a fund of £8,152 was collected in aid of the refugees in Egypt. The sufferers from the fighting in the Balkan Peninsula, in 1876, benefited to the extent of £17,250, and the victims of the famine in China in 1889 by £32,654. The bald record is eloquent in itself, containing as it does such entries as "Chios Earthquake, £24,750;' "Famine in Iceland, £7,867;" "Cholera in Spain £4,243;" and "Fire at Salonica, £3,740." Could anything be more suggestive of the charity which is blind to the prejudices of race or creed?

A glance at the history of Mansion House Funds proves, indeed, beyond doubt, that the springs of charity are not drying up. There are times when, owing to the depression of trade or other circumstances of a temporary character, the ability to contribute to the relief of distress is narrowed. But the will is always there, and as time goes on the growth in the number of contributors is no less marked than the increase in the aggregate of the sums raised.

